# Polymer-based antibody mimetics (iBodies) target human PD-L1 and function as a potent immune checkpoint blocker

**DOI:** 10.1016/j.jbc.2024.107325

**Published:** 2024-04-27

**Authors:** Mohammad Reza Zamani, Martin Hadzima, Kristýna Blažková, Vladimír Šubr, Tereza Ormsby, Javier Celis-Gutierrez, Bernard Malissen, Libor Kostka, Tomáš Etrych, Pavel Šácha, Jan Konvalinka

**Affiliations:** 1 Faculty of Science, Department of Cell Biology, Charles University, Prague, Czech Republic; 2 Institute of Organic Chemistry and Biochemistry, Czech Academy of Sciences, Prague, Czech Republic; 3 Faculty of Science, Department of Organic Chemistry, Charles University, Prague, Czech Republic; 4 Department of Biomedical polymers, Institute of Macromolecular Chemistry, Czech Academy of Sciences, Prague, Czech Republic; 5 Centre d’Immunologie de Marseille-Luminy, Aix-Marseille Université, INSERM, CNRS, Marseille, France; 6 Faculty of Science, Department of Biochemistry, Charles University, Prague, Czech Republic

**Keywords:** PD-1, PD-L1, immune checkpoint, HPMA copolymer, antibody mimetic, T-cell, inhibitor, immunotherapy, immunosuppression, tumor immunology

## Abstract

Immune checkpoint blockade (ICB) using monoclonal antibodies against programmed cell death protein 1 (PD-1) or programmed death-ligand 1 (PD-L1) is the treatment of choice for cancer immunotherapy. However, low tissue permeability, immunogenicity, immune-related adverse effects, and high cost could be possibly improved using alternative approaches. On the other hand, synthetic low-molecular-weight (LMW) PD-1/PD-L1 blockers have failed to progress beyond *in vitro* studies, mostly due to low binding affinity or poor pharmacological characteristics resulting from their limited solubility and/or stability. Here, we report the development of polymer-based anti-human PD-L1 antibody mimetics (α-hPD-L1 iBodies) by attaching the macrocyclic peptide WL12 to a *N*-(2-hydroxypropyl)methacrylamide copolymer. We characterized the binding properties of iBodies using surface plasmon resonance, enzyme-linked immunosorbent assay, flow cytometry, confocal microscopy, and a cellular ICB model. We found that the α-hPD-L1 iBodies specifically target human PD-L1 (hPD-L1) and block the PD-1/PD-L1 interaction *in vitro*, comparable to the atezolizumab, durvalumab, and avelumab licensed monoclonal antibodies targeting PD-L1. Our findings suggest that iBodies can be used as experimental tools to target hPD-L1 and could serve as a platform to potentiate the therapeutic effect of hPD-L1-targeting small molecules by improving their affinity and pharmacokinetic properties.

Immunotherapy has become a pillar of cancer treatment along with chemotherapy, radiation, and targeted therapies. This therapeutic approach boosts patient’s own immune system to fight cancer cells (1). In particular, immune checkpoint blockade (ICB) using antibodies targeting programmed cell death-1 (PD-1/CD279) and programmed death-ligand 1 (PD-L1/CD274/B7-H1) has shown remarkable benefits for patients with advanced malignancies (1, 2).

PD-1 is mainly expressed in activated T cells and sustained levels of PD-1 are observed in T cells during chronic infection and cancer (3). PD-L1 is naturally expressed in antigen-presenting cells and a variety of tissues (4). The PD-1/PD-L1 interaction mitigates/dampens T-cell activity to avoid uncontrolled immune response and inflammation (5). However, many cancers use this phenomenon to their advantage. Overexpression of PD-L1 in tumor cells initiates inhibitory signals in activated T cells (6) leading to T cell anergy, apoptosis, exhaustion, and subsequent tumor evasion (7).

Clinical studies have shown that inhibiting the PD-1/PD-L1 interaction enhances anti-tumor immunity and survival (8). Both PD-1-targeting monoclonal antibodies (mAbs; nivolumab, pembrolizumab, cemiplimab, and dostarlimab) and PD-L1-targeting mAbs (atezolizumab, avelumab, and durvalumab) have demonstrated therapeutic efficacy in various cancer types and achieved U.S. Food and Drug Administration (FDA) approval (8).

Antibody-based ICB is one of the most effective treatments available today. Nevertheless, ongoing efforts in both basic research and pharmaceutical companies are aiming at developing alternative approaches, seeking potential enhancements to certain aspects of antibody-based ICB, such as low tissue permeability of antibodies (9), their immunogenicity (*e.g.*, atezolizumab) (10), *in vivo* cleavage by proteases (11), immune-related adverse effects, poor pharmacokinetics (12, 13), and high manufacturing costs (14). Furthermore, it is hypothesized that a functional Fc region in α-PD-L1 antibodies can lead to the depletion of PD-L1+ T cells in the tumor microenvironment (TME), which is not favorable (15, 16). Among the therapeutic PD-L1-targeting mAbs, only avelumab has Fc-FcγR binding ability.

One way to address the shortcomings of antibody-based therapeutics is to develop fully synthetic low-molecular-weight (LMW) PD-1/PD-L1 blockers. Although many LMW PD-1/PD-L1 blockers have been patented by Bristol-Myers Squibb (BMS) and Aurigene Discovery Technologies (17), none have been clinically approved yet, and very limited data on their *in vitro* or *in vivo* activity are available. The few published studies indicate that LMW blockers have a lower ability to disrupt the PD-1/PD-L1 interaction in both cell-free and cellular models, compared to therapeutic antibodies (18, 19, 20).

An alternative approach is to develop antibody mimetics, synthetic compounds with antigen-binding ability similar to or greater than that of native antibodies, such as monobodies, nanobodies, affimers, aptamers, or knottins (21). PD-L1-targeting antibody mimetics with high stability and affinity have already been designed and studied (22, 23).

Recently, we developed antibody mimetics called iBodies. They consist of a bio- and immune-compatible hydrophilic *N*-(2-hydroxypropyl)methacrylamide copolymer (pHPMA) backbone decorated with target-specific LMW ligands, as well as fluorescent molecules and biotin for facile imaging and isolation of the protein of interest. Our previous studies have shown that iBodies improve the solubility, stability, and functional affinity (avidity) of LMW compounds, thus making them effective tools to target, inhibit, visualize, and isolate proteins (24, 25, 26). In particular, one study demonstrated an improved anti-tumor effect of an HPMA-linear peptide conjugate targeting mouse PD-L1 compared to the free peptide in syngeneic BALB/c mice bearing 4T1 tumors (27).

In the present study, we describe the development of anti-human PD-L1 iBodies (α-hPD-L1 iBodies) using a hPD-L1-specific macrocyclic peptide called WL12. This compound combines the general advantages of macrocyclic peptides over their linear counterparts (28) with the presence of a single primary amine for facilitated chemical conjugation to pHPMA (29).

To the best of our knowledge, it is the first study comparing a fully synthetic, non-GMO (genetically modified organism) and non-animal origin α-hPD-L1 reagent that specifically targets hPD-L1 and effectively blocks the PD-1/PD-L1 interaction *in vitro*, with an efficacy similar to the current FDA-approved therapeutic α-hPD-L1 antibodies atezolizumab, avelumab, and durvalumab. This versatile, convenient, and biocompatible alternative may potentially improve some of the shortcomings of current LMW compounds and monoclonal antibodies.

## Results

### Synthesis of WL12 and iBodies

**Figure 1:**
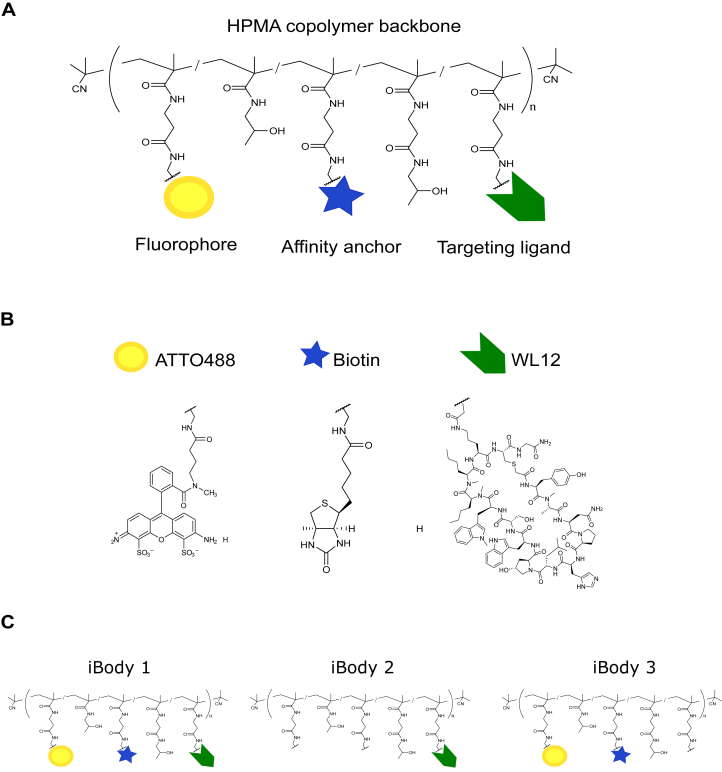
**Chemical structure of the *N*-(2-hydroxypropyl)methacrylamide (HPMA) copolymer carrier called iBody and its functional ligands.***A*, general design of iBodies. iBodies can carry various ligands, for example a fluorophore (*yellow*), an affinity anchor (*blue*) and a targeting ligand (*green*). *B* and *C*, anti-human PD-L1 iBodies (α-hPD-L1 iBodies) were synthesized by decorating HPMA copolymers (pHPMA) with the PD-L1-targeting ligand WL12 (iBody 1 and iBody 2), a biotin affinity anchor (iBody 1 and iBody 3) and an ATTO488 fluorophore (iBody 1 and iBody 3).

Synthesis and characterization of WL12 as well as a brief reaction scheme for polymer precursor and iBody synthesis are described and illustrated in Experimental procedures and Supplementary methods. The iBodies were synthesized by employing aminolytic reaction between reactive TT groups of polymer precursors P1 and P2 with amino groups of the fluorophore, affinity anchor, and targeting ligand. The schematic structure of iBodies is depicted in Figure 1. The aminolytic reactions proceed almost quantitatively and all iBodies contained sufficient number of ligands. The slightly lower content of ATTO488 and biotin in iBody1 in comparison to iBody 3 is caused by the overall higher number of ligands bound to iBody 1.

### iBodies tightly bind to recombinant hPD-L1

One of the general drawbacks of PD-L1-targeting LMW compounds, including WL12, is their relatively low binding affinity compared to antibodies. We assessed the specificity and functional binding affinity (avidity) of the iBodies to immobilized recombinant human PD-L1 (rhPD-L1 using *in vitro* assays such as ELISA, and surface plasmon resonance (SPR).

**Figure 2:**
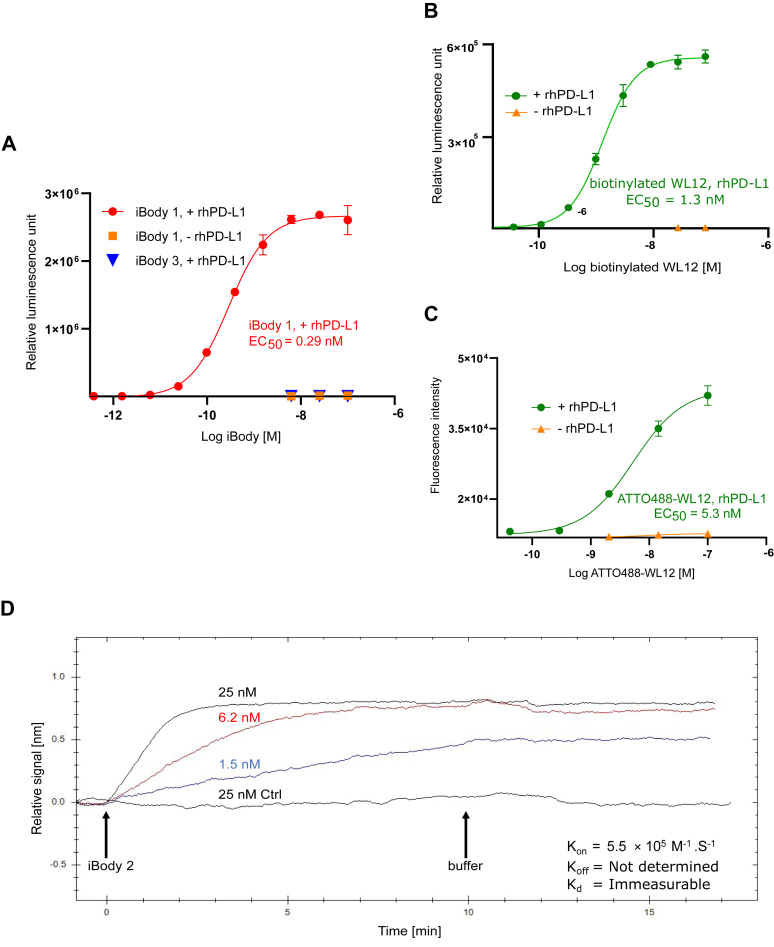
**Binding characteristics of α-PD-L1 iBodies and WL12 to immobilized rhPD-L1.***A–C*, determination of EC_50_ for iBody 1 and WL12 in a sandwich ELISA. Dilution series of iBody 1 (biotinylated and carrying WL12), iBody 3 (negative control, lacking the PD-L1-targeting ligand), biotinylated WL12 and ATTO488-WL12 were incubated in the presence or absence of 100 ng of directly immobilized rhPD-L1. The interaction for biotinylated WL12 and iBodies was detected by addition of Neutravidin-HRP and subsequent measurement of relative luminescence, whereas for ATTO-WL12, the detection was carried out by measuring the fluorescence intensity. EC_50_ was calculated using a 4-parameter logistic function in GraphPad Prism 9.0.0. *D*, kinetic parameters of the interaction of iBody 2 with immobilized rhPD-L1. SPR was performed using three concentrations of iBody 2 (carrying the PD-L1-targeting ligand only) in the presence of immobilized rhPD-L1. The negative control (25 nM Ctrl, *black*) contains the highest concentration of iBody 2 in the absence of rhPD-L1. Kinetic parameters were calculated by fitting the curves using a one-to-one model in TraceDrawer v.1.5 software. Data are representative of three independent experiments.

We evaluated the specificity and binding potency of iBody 1 (carrying WL12, ATTO488, and biotin moieties) and iBody 3 (negative control; lacking WL12 but carrying ATTO488 and biotin moieties) (Fig. 1 and Table 1) to immobilize rhPD-L1 with sandwich ELISA (Fig. 2*A*). In this setup, iBody 1 showed high specificity and binding potency towards rhPD-L1 with an EC_50_ of 0.29 nM (95% CI = 0.24–0.34), which was comparable with published EC_50_ values for durvalumab (∼0.32 nM) and atezolizumab (0.22 ∼ 0.32 nM) using indirect ELISA (http://en.atagenix.com/product_detail-23647.html) (30). We also analyzed the interaction between fluorescently labeled (ATTO488-WL12) as well as biotinylated ligand (biotinylated-WL12) with rhPD-L1 in a similar ELISA setup. WL12 shows lower molar potency of target binding when compared to iBody 1 and therapeutic antibodies (Fig. 2*B* EC_50_ = 5.3 nM, 95% CI = 3.6–8.3; Fig. 2*C* EC_50_ = 1.3 nM, 95% CI = 1.1–1.4).

**Table 1:** The characterization and composition of anti-hPD-L1 iBodies

Polymer conjugate	iBody 1	iBody 2	iBody 3
Target	hPD-L1	hPD-L1	No Target (Negative Control)
Mw [kDa]	110	85	74
Mw’ [kDa]	86	81	73
Ð	1.16	1.13	1.06

	Moiety	No. per polymer	Moiety	No. per polymer	Moiety	No. per polymer
Targeting ligand	WL12	5.40	WL12	8.28	-	-
Fluorophore	ATTO488	2.4	-	-	ATTO488	3.6
Affinity anchor	Biotin	7.50	-	-	Biotin	11.9

Mw, weight-average molecular weights; Mw’, Mw of precursor polymer, increased by the sum of apparent molecular weights of all conjugated ligands; Ð, dispersity; No. per polymer, an average number of conjugated ligands per molecule of HPMA polymer carrier.

We performed SPR to verify the binding ability and assess the reaction kinetics of iBody 2 (carrying only WL12; Fig. 1 and Table 1) with immobilized rhPD-L1. Since the dissociation/off rate of the iBody 2 is slow (Fig. 2*D*), precise determination of k_off_ and *K*_*d*_ using SPR is impossible. However, the overall binding strength of iBody 2 to PD-L1 seems to be in agreement with the ELISA EC_50_ and comparable to published *K*_*d*_ values for atezolizumab, avelumab, and durvalumab (1.75 nM, 0.04 nM, and 0.66 nM, respectively) (31). In another study, a modified WL12 (copper-64 radiolabeled NOTA-WL12) has been tested by the SPR method, showing weaker affinity (*K*_*d*_ = 3 nM) (32) as compared to iBody 2 and therapeutic antibodies due to faster dissociation.

Unlike most LMW compounds and antibodies tested by SPR, the very slow dissociation rate between iBody 2 and rhPD-L1 could indicate a strong avidity effect.

### iBodies detect and visualize membrane-bound hPD-L1

**Figure 3:**
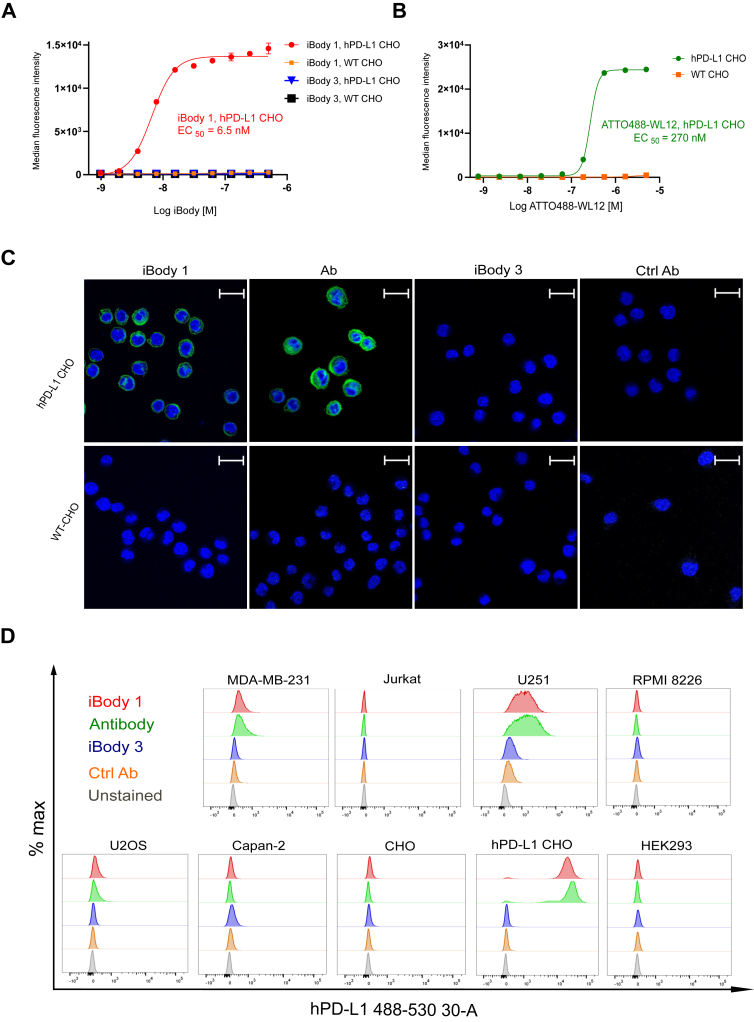
**iBodies bind to membrane-bound hPD-L1.***A*, flow cytometry analysis of iBody 1 (carrying WL12, biotin and ATTO488) and iBody 3 (mock polymer, carrying biotin and ATTO488) binding to hPD-L1-transfected and wild-type (hPD-L1 and WT) CHO cells. Cells were measured on a BD LSRFortessa flow cytometer, gated for single cells, and analyzed with FlowJo 10.4.1(Fig. S4). EC_50_ was calculated from the median fluorescence intensity (MFI) using a 4-parameter logistic function in GraphPad Prism 9.0.0 (representative of two experiments). *B*, flow cytometry analysis of ATTO488-WL12 using the same cell lines and methods (representative of two experiments). *C*, visualization of hPD-L1 expression on hPD-L1-transfected and wild-type CHO cells. Cells were fixed with 2% formaldehyde (30 min, RT), blocked with 1% BSA (2 h, RT), and stained with 10 nM of iBody 1 or iBody 3 or Alexa Fluor 488-conjugated α-hPD-L1 antibody or the control antibody (Ctrl Ab; Alexa Fluor 488-conjugated mouse IgG1 kappa, clone: P3.6.2.8.1) were added directly to seeded CHO cells transfected or untrasnfected with PD-L1 (1 h, RT). Cells were washed, nuclei were stained with 2 μg/ml Hoechst 33342 and visualized using a Zeiss confocal microscope. Scale bar = 20 μm. The figure shows representative data out of two experiments. *D*, detection of hPD-L1 expression on cancer cell lines. A panel of cancer cell lines was stained with 100 nM iBody 1 (*red*), 100 μl of 1:100 FITC-conjugated α-PD-L1 antibody (clone 29E.2A3, green) or 100 nM iBody 3 (*blue*) and 15 ug/ml control antibody (Ctrl Ab; mouse FITC-conjugated IgG2b, clone MPC-11, *yellow*) or without staining (*grey*). Adherent cells were trypsinized and washed alongside with suspension cells and stained for 30 min at 4 °C. Cells were washed, measured on a BD LSRFortessa flow cytometer, and gated for single cells (Fig. S7). The data were analyzed with FlowJo 10.4.1.

Taking advantage of the versatility of our iBodies, we determined the specific binding of α-hPD-L1 iBodies to hPD-L1 in the cellular context by tracking the ATTO488 fluorophore moiety using flow cytometry and confocal microscopy. First, using a dilution series of iBody 1 and iBody 3, we observed specific and concentration-dependent binding of iBody 1 to hPD-L1-transfected Chinese hamster ovary (CHO PD-L1) cells (EC_50_ = 6.6 nM, 95% CI = 5.9–7.3) (Fig. 3*A*) compared to iBody 3 or untransfected Chinese hamster ovary cell line (CHO). Later, we also tested the staining efficiency of the WL12 conjugated with ATTO488 fluorophore (ATTO488-WL12). In contrast to iBody 1, staining of CHO PD-L1 cells with ATTO488-WL12 was significantly less efficient than with iBody 1 (EC_50_ = 270 nM, 95% CI = 250–280) (Fig. 3*B*).

Then, we visualized membrane-bound hPD-L1 on CHO PD-L1 cells with confocal microscopy, using 10 nM of iBody 1 or iBody 3 and commercial Alexa Fluor 488-conjugated α-hPD-L1 antibody or an isotype control (Fig. 3*C*). Obtained images indicate that iBody 1 stains hPD-L1 in a similar pattern as α-hPD-L1 antibodies under identical imaging settings.

Next, we selected a panel of cancer cell lines from our laboratory cell bank to compare the detection of hPD-L1 using iBodies and a highly potent commercial antibody (Fig. 3*D*). The results showed that iBody 1 has similar sensitivity and specificity in staining of cell lines as the α-hPD-L1 antibodies with U251 glioblastoma and MDA-MB-231 breast cancer cell lines showing PD-L1 expression as expected. Overall, the results indicate that iBody 1 targets membrane hPD-L1 with high efficiency, comparable to commonly used experimental α-PD-L1 antibodies.

### iBodies are potent immune checkpoint blockers

The biological potencies of PD-1/PD-L1 blockers can be studied *in vitro* using T cell-based assays (33). These assays typically consist of a co-culture model of PD-L1-expressing cells and of T cells that have been either subjected to an activation signal (TCR/CD3) to induce the expression of PD-1 or that constitutively express PD-1 (34). Blockade of PD-1/PD-L1 engagement relieves the inhibitory signals and leads to elevated T cell proliferation and activation in the form of cytokine production (*e.g.*, interleukin 2, IL-2, and interferon γ, IFN-γ), upregulation of membrane markers (*e.g.*, CD69 and CD25), and alteration of PD-1-related downstream signaling pathways (35).

**Figure 4:**
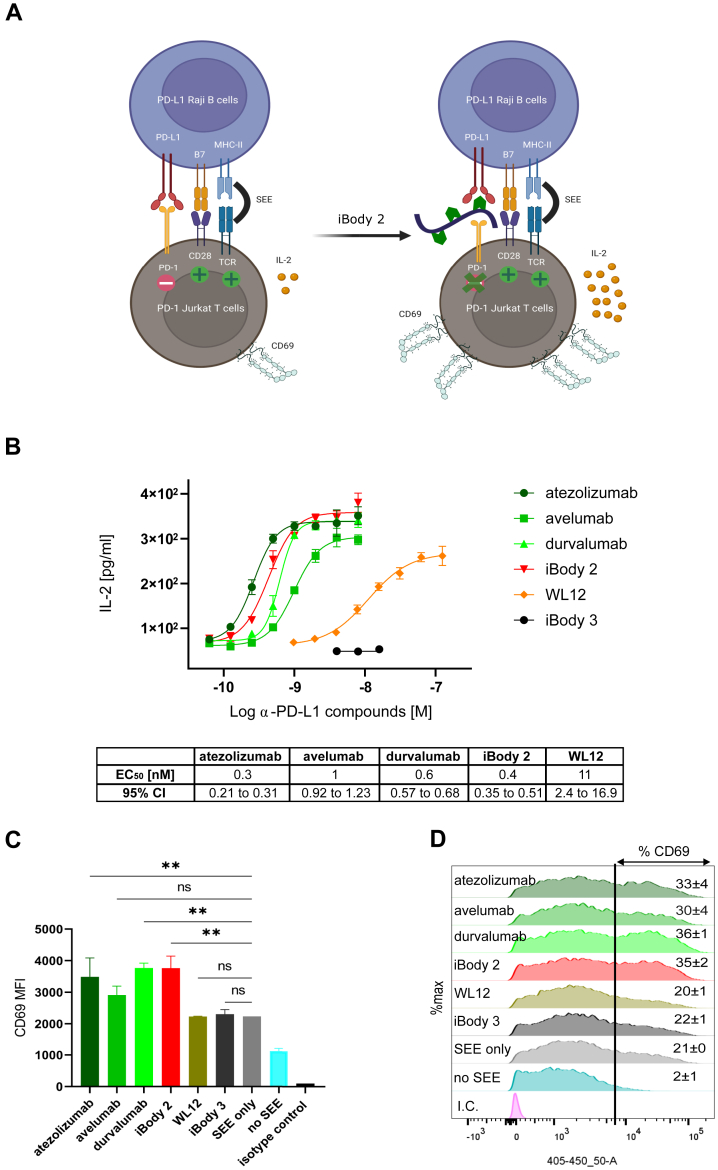
**α-hPD-L1 iBodies restore activation of PD-1/PD-L1-suppressed Jurkat T cells.***A*, schematic illustration of the cellular model for *in vitro* evaluation of α-PD-L1 compounds (created in biorender.com). *B*, PD-L1-overexpressing Raji B cells were pre-incubated with a dilution series of α-PD-L1 compounds in the presence of 25 pg/ml SEE for 1 h and then co-cultured in a 1:1 ratio with PD-1-overexpressing Jurkat T cells for 24 h. Supernatants were collected, and IL-2 levels were measured using ELISA. EC_50_ was calculated using a 4-parameter logistic function in GraphPad Prism 9.0.0. Data are representative of five independent experiments performed in duplicates, with error bars indicating the standard deviation. *C*, PD-L1-overexpressing Raji B cells were pre-incubated with 2 nM of α-PD-L1 compounds in the presence of 25 pg/ml SEE for 1 h and then co-cultured in a 1:1 ratio with PD-1-overexpressing Jurkat T cells for 18 h. Cells were collected and stained with BV421-conjugated anti-human CD69 antibody. The isotype control antibody (100 ng) was used to stain the iBody 2-treated sample. Cells were measured with a BD LSRFortessa instrument (Fig. S9). Data are represented as median fluorescence intensity (MFI). Error bars indicate the standard deviation. *D*, histograms indicate the percentage of CD69-expressing cells (gated above the basal CD69 expression in the absence of SEE; no SEE). Data were analyzed with FlowJo 10.4.1 and are representative of three independent experiments performed in duplicates. MFI comparison was carried out using a one-way ANOVA test in GraphPad Prism 9.0.0. ∗∗ indicates *p*-value < 0.01; ns = not significant.

Since our α-hPD-L1 iBodies were potent binders of hPD-L1, we sought to evaluate their functional blocking effect and to compare it with WL12 and the existing FDA-approved therapeutic antibodies using a published cellular model (35) (Fig. 4*A*). In this model, hPD-1-overexpressing Jurkat T cells and hPD-L1-overexpressing Raji B cells are co-cultured in the presence of the superantigen staphylococcal enterotoxin E (SEE). The interaction of PD-1 with PD-L1 suppresses T-cell activation. In the presence of a PD-1/PD-L1 blocker, the PD-1-mediated inhibition is diminished, resulting in restored T-cell activation (36).

To assess T cell activation, we analyzed both IL-2 levels in cell culture supernatants with ELISA (Fig. 4*B*) and membrane CD69 expression with flow cytometry (Fig. 4, *C* and *D*). As illustrated in Figure 4*B*, iBody 2 blocks PD-1/PD-L1 engagement in a concentration-dependent manner and restores T cell activation with high potency (EC_50_ = 0.4 nM, 95% CI = 0.35–0.51), while the control iBody 3 (mock polymer, carrying biotin and ATTO488) has no significant effect. This potency is significantly higher than that of free WL12 (EC_50_ = 11 nM, 95% CI = 2.4–16.9) and comparable to therapeutic antibodies atezolizumab (EC_50_ = 0.3 nM, 95% CI = 0.21–0.31), avelumab (EC_50_ = 1 nM, 95% CI = 0.92–1.23), and durvalumab (EC_50_ = 0.6 nM, 95% CI = 0.57–0.68). Results obtained from determining the surface CD69 expression agreed with IL-2 production. As shown in Figure 4, *C* and *D*, using the same cellular model, a substantial increase in the overall expression of CD69 occurred in samples treated with 2 nM of each of the PD-1/PD-L1 blockers atezolizumab, durvalumab, and iBody 2, as compared with non-treated samples, while avelumab and WL12 did not show significant differences.

To summarize, iBodies function as a potent *in vitro* immune checkpoint blocker *via* targeting PD-L1.

## Discussion

This work presents the development of HPMA-based antibody mimetics, called α-hPD-L1 iBodies, targeting human PD-L1. Several mAbs targeting PD-1 and PD-L1 have achieved FDA approval for the treatment of cancer (37, 38), but they suffer from a number of drawbacks, such as low tumor tissue permeability, potential immunogenicity associated with the protein nature (10, 39, 40), immune-related adverse effects (41), low modularity, low-temperature stability, development of resistance and high cost (one checkpoint blockade treatment for a single patient costs approximately $1 million (14)). On the other hand, small molecules, including WL12, have lower binding affinity to human PD-L1 compared to antibodies (32) and are poor *in vitro* blockers of the PD-1/PD-L1 interaction with limited ability to restore T cell activation (20).

We have previously shown that decorating HPMA copolymers with target-specific small-molecule ligands generates a biocompatible iBody with high aqueous solubility, high thermostability, and avidity (24, 25, 42). We, therefore, set to develop α-hPD-L1 iBodies using a previously described low-molecular-weight macrocyclic peptide WL12. This ligand is specific for human PD-L1, has a relatively high affinity compared to other published peptides, and possesses a single primary amine for facile chemical conjugation with HPMA copolymer. Despite WL12’s low potency and inability to interfere with the therapeutic effects of α-PD-L1 antibodies in a patient, a radiolabeled WL12 for imaging was developed to determine the PD-L1 expression in non-small cell lung cancer (28).

Generally, tethering of low-molecular-weight ligands to a sterically demanding polymeric random coil comes with a risk of affecting the ligand-target interaction. However, we anticipated that WL12 would be compatible with this approach due to its relatively large size and stable cyclic structure. Furthermore, the binding affinity of WL12 to PD-L1, as determined by SPR, was found to be around 3 nM – nearly 200 times better than the binding affinity of PD-1 (43) or of the linear peptide PPA-1 to PD-L1 (44). Interestingly, when PPA-1 was incorporated into doxycycline-containing nanoparticles or pHPMA-conjugates, it displayed promising anti-tumor properties (45).

Our results indicate that conjugation of WL12 to iBodies leads to an increased binding affinity (Fig. 2, *A–D*) to a sub-nanomolar level that is comparable to therapeutic antibodies published in the literature (46). We observed several-fold improvement in both cell-free and cellular assays as well as in the biological potency of iBodies that goes beyond the number of conjugated ligand moieties. This could be associated with the general characteristics of iBodies, including increased ligand solubility due to pHPMA hydrophilicity (47) and generation of a strong avidity effect due to multivalency (48). The avidity effect hypothesis is supported by a very low dissociation rate between iBodies and rhPD-L1 detected by SPR. The importance of avidity effects for blocking PD-1/PD-L1 interaction was also observed by Bu *et al.* (49). They showed that conjugation of an α-hPD-L1 antibody to dendrimers improved PD-L1 binding by up to an order of magnitude which correlated with the rescue of IL-2 production in a Jurkat co-culture model and increased tumor accumulation.

It would be intriguing to discuss the binding site of the iBody to the rhPD-L1. However, we were not able to get corresponding structural data and thus could only speculate based on the published structure of the WL12-PD-L1 complex. According to (18), WL12 binds to a similar binding pocket as PD-1 and the hydrophobic interactions are critical for the binding affinity. The binding of the peptide partially overlaps the binding site for the mAb currently used for the therapy (atezolizumab, avelumab, or durvalumab) and the peptide itself mimics the hydrophobic interactions of the complementarity-determining regions of the antibodies. The authors claim that the peptide uses only a part of the interaction surface and that further modification leading to binding improvement is still possible. We speculate that the increased efficiency of iBodies is caused by the fact that local steric hindrance formed around iBody-PD-L1 interface by the polymeric scaffold would enhance their ability to disrupt a PD-1/PD-L1 interaction.

The efficacy and side effects of mAbs are connected to their half-life *in vivo*. iBodies could be designed using cleavable linkers in the polymer backbone (27), or by modifying their molecular weight to guarantee optimal pharmacokinetics (50, 51) and thus mitigate potential overactivation of the immune system (52).

From the point of view of potential clinical applications, the iBodies approach may have additional advantages, such as improving rather short half-lives of proteins, peptides, and peptidomimetics (53) by reducing *in vivo* proteolytic cleavage thanks to a sterically demanding and nonreactive backbone. Additionally, the relatively large yet adjustable size of the iBodies may also improve the poor pharmacokinetics of the α-PD-L1 small molecule compounds as a result of reducing their kidney excretion and increasing tumor accumulation *via* the EPR effect (54).

Consistent with our approach, a recent study using a pHPMA-linear peptide conjugate against mouse PD-L1 showed promising *in vivo* ICB and anti-tumor effects in a syngeneic mouse tumor model (27). This conjugate was tested in combination with preceding immunogenic chemotherapy and proved to increase the effectiveness of such treatment. The authors assume that the improved anti-tumor effect of their pHPMA conjugate is associated with multivalency and subsequent PD-L1 crosslinking that causes its lysosomal degradation, along with an enhanced permeability and retention (EPR) effect arising from large polymer size (55). This study (27) provides extensive *in vivo* data that supports the therapeutic potential of PD-L1-targeted HPMA-based conjugates.

Our study focused on a comprehensive *in vitro* characterization of iBodies targeting human PD-L1 in comparison with the FDA-approved and highly potent experimental monoclonal antibodies (clone 29E.2A3). Moreover, we showed that iBodies performed with high specificity and sensitivity in experimental detection and visualization of hPD-L1 using flow cytometry and confocal microscopy comparable to that of the aforementioned mAbs. Given the versatility and ease of manufacturing of iBodies, a library of α-hPD-L1 iBodies with a wide variety of different characteristics could be developed and preclinically tested in a humanized mouse model. This will be the subject of our future investigations.

## Conclusion

In summary, we developed α-hPD-L1 iBodies and characterized them *in vitro*. To the best of our knowledge, these are the first fully synthetic and biocompatible α-hPD-L1 reagents with no animal or GMO sources that target hPD-L1 and block the hPD-L1/hPD-1 interaction with potency comparable to that of existing FDA-approved α-PD-L1 therapeutic antibodies. This versatile approach opens new possibilities for further development of pHPMA-based α-hPD-L1 antibody mimetics in order to address the shortcomings of LMW compounds and monoclonal antibodies.

## Experimental procedures

### Synthesis of WL12

ClAc-Tyr-MeAla-Asn-Pro-His-Leu-Hyp-Trp-Ser-Trp(Me)-MeNle-MeNle-Orn-Cys-Gly-NH2 was synthesized by SPPS manually using standard Fmoc chemistry protocols, HATU/DIPEA coupling conditions and Rink Amide MBHA resin support (0.2 mmol scale; 5 eq. amino acid excess). Fmoc groups were removed using 20% piperidine in DMF. Coupling modifications were introduced for Cys and ClAcOH (HATU/collidine); longer reaction times were employed for the deprotection of secondary amines and coupling to secondary amines. A detailed description of the synthesis and conjugation of ATTO488 fluorophore can be found in Supporting information. Peptide amino acid side chains were deprotected, and the peptide was cleaved off the resin with a 95:2.5:2.5 mixture of TFA:H_2_O:TIS for 1 h at room temperature. The residue was triturated with diethyl ether to obtain crude peptide, which was used in the next step without further purification. Cyclization was performed according to a published procedure (56). The crude peptide was dissolved in 1:1 MeCN:0.1 M NH_4_OAc (300 ml), and the pH was carefully adjusted to 8.5 to 9.0 using aq. 1 M NaOH. The solution was allowed to stand without stirring for 18 h. Solvents were subsequently removed by lyophilization. The product was purified by reverse phase C18 HPLC (gradient 5–70% MeCN in water + 0.1% TFA) to obtain WL12 as a TFA salt (70 mg, 18%). The identity of the compound was verified by MALDI MS: [M + H]+ 1882.

### Synthesis of polymer precursors

Monomers *N*-(2-hydroxypropyl)methacrylamide (HPMA) and 3-(3 methacrylamidopropanoyl)thiazolidine-2-thione (Ma-β-Ala-TT) and chain transfer agent S-2-cyano-2-propyl S′-ethyl trithiocarbonate were synthesized as previously described (57, 58, 59). HPMA copolymer precursors poly(HPMA-co-Ma-β-Ala-TT) (P1 and P2) were prepared by reversible addition-fragmentation chain transfer (RAFT) copolymerization of HPMA and Ma-β-Ala-TT. Detailed description of the synthesis can be found in Supporting information. Characteristics of synthesized precursors are summarized in Table S1.

### Synthesis of iBodies

The copolymer precursors were reacted with appropriate components in DMSO in the presence of DIPEA. For iBody 1 precursor P1 reacted with NH_2_-PEG_11_-biotin, ATTO488 and WL12. For iBody 2 precursor P2 reacted with WL12. And for iBody 3 precursor P1 reacted with NH_2_-PEG_11_-biotin, and with ATTO488. Detailed description of the synthesis can be found in Supporting information. All iBodies were characterized by size exclusion chromatography for molecular weight and dispersity. The content of the individual components was analyzed spectrophotometrically or with amino acid analysis as in the case of the WL12. Detailed parameters of all analytical approaches are described in Supporting information. The yield of each iBody was 8 mg.

### Cloning, expression, and purification of recombinant human PD-L1 extracellular domain

DNA encoding extracellular domain (residues 19–239) of human PD-L1 was amplified by PCR from pCMV3-full-length human PD-L1 plasmid (Sino Biological Inc. cat# HG10084-CH), and subcloned into pTT28 vector (National Research Council of Canada) between *Nhe*I and *AgeI* restriction sites. The vector pTT28ectoPDL1 has an N-terminal secretion leader sequence and a C-terminal His8-tag sequence for high-yield protein expression and secretion in human embryonal kidney cells (HEK293-6E) and facile purification. Briefly, HEK293-6E cells were grown in freestyle 293 medium (Gibco) and transiently transfected with 2 μg/ml pTT28ectoPDL1 with 5:1 ratio of polyethylenimine to DNA. The medium was collected 7 days after the transfection, and the recombinant extracellular domain of His-tagged human PD-L1 was purified on nickel affinity chromatography using Ni-NTA Agarose (Sigma). The protein was eluted with 50 mM Tris/HCl, pH 8.0, 300 mM NaCl, 100 mM imidazole, and subsequently dialyzed to remove imidazole. SDS-PAGE was carried out to assess the protein purity. The final concentration and yield of the purified protein were determined by Bradford assay, giving 2.9 mg/ml and 5.8 mg, respectively.

### ELISA

A black flat-bottom 96-well plate (Nunc MaxiSorp, Thermo Fisher Scientific) was coated with 100 μl/well recombinant extracellular hPD-L1 (1 ng/μl) in Tris-buffered saline (TBS 150 mM NaCl, 50 mM Tris-HCl, pH 7.5) for 1 h at RT. Following three washes with 200 μl TBST (TBS with 0.05% Tween-20 detergent), the wells were blocked with 200 μl 1:4 diluted casein (SDT, cat# CBC1) in TBS overnight at 4 °C with shaking. After three washes, 100 μl of dilution series of biotinylated WL12, iBody 1 and iBody 3 (control) in TBST′ (TBS with 0.1% Tween-20) were added to the corresponding wells for 1 h at RT with shaking. Finally, following three washes, 100 μl (1 ng/well) neutravidin-HRP (1 h, RT, TBST′) was added to the wells for detection of the bound iBodies. Three additional wash steps were performed. Immediately after the addition of 200 μl HRP substrate, chemiluminescence was measured at 428 nm using a TECAN Infinite M1000 plate reader (TECAN). Values were shown as relative luminescence units (RLU). ATTO488-WL12 ELISA was performed using a principally similar experimental procedure. Briefly, wells were coated with 100 ng/well recombinant extracellular hPD-L1 in TBS for 1 h at RT, following three washes with 300 μl TBST. Then, the wells were blocked with 300 μl 1:4 diluted casein in TBS overnight at 4 °C with shaking. After three washes, 100 μl of dilution series of ATTO488-WL12 in TBST′ were added to the corresponding wells for 1 hat RT with shaking. Finally, following four washes with TBST, two additional washes were done with distilled water to remove the buffer and tween. The wells were emptied and ATTO488 fluorescence intensity was measured using a TECAN Infinite M1000 plate reader. EC50 was calculated using a 4-parameter logistic function in GraphPad Prism 9.0.0.

### Surface plasmon resonance spectroscopy

A four-channel SPR sensor instrument manufactured by the Institute of Photonics and Electronics (IPE), Prague, was used to perform SPR measurements. First, gold-coated SPR chips (also manufactured by the IPE) were functionalized by incubating in a pure ethanol mixture of HS-(CH_2_)_11_-PEG_6_-O-CH_2_-COOH alkanethiols and HS-(CH_2_)_11_-PEG_4_-OH (molar ratio 3:7) with a final concentration of 0.2 mM for 1 h at 37 °C. The functionalized chip was mounted to the SPR sensor instrument. Then, the carboxylic terminal groups on the sensor surface were activated by injecting a 1:1 mixture of 100 mM *N*-hydroxysuccinimide and 400 mM 1-ethyl-3-(3-dimethylaminopropyl)-carbodiimide hydrochloride in deionized water for 5 min at a flow rate of 30 μl/min. Next, neutravidin solution (1 ml of 0.02 mg/ml in 10 mM sodium acetate buffer, pH 5.0) was injected into all channels to bind to the carboxylic terminal groups. Afterward, high ionic strength phosphate-buffered saline (PBS; 0.5 M NaCl, 2.7 mM KCl, 13.5 mM K_2_HPO_4_, 5 mM NaH_2_PO_4_.2H_2_O, pH 7.2) solution was run through the channels to remove the unbound neutravidin molecules, followed by blocking unreacted carboxyl groups using 1 M ethanolamine (Biacore). A 1.5 ml solution of 50 nM biotinylated anti-histidine tag iBody (anti-His iBody (42)) in TBST″ (TBS with 0.01% Tween-20) was run through all channels to bind to the neutravidin. Next, 1.5 ml (8.25 μg/ml) His-tagged extracellular rhPD-L1 in TBST″ was injected into three channels to be immobilized by anti-His iBodies (one channel was kept as negative control). Finally, three concentrations of iBody 2 (25 nM, 6.2 nM, and 1.5 nM) and one high concentration of iBody 2 (25 nM for the control channel) in TBST″ were injected in the corresponding channels for a few minutes (K_on_; association constant), followed by injection of buffer alone (K_off_; dissociation constant) into all channels. The real-time interactions were monitored with SPR_UP software and the kinetic parameters were extracted by fitting the iBody 2 binding curves in TraceDrawer v.1.5.

### Cell culture

All cell lines were grown and maintained at 37 °C, 5% CO_2_, >95% humidity, and ambient oxygen levels in a cell culture incubator. All media were supplemented with 10% fetal bovine serum (FBS-Gibco). Cells lines were obtained and cultured as follows: MDA-MB-231 (kindly provided by Dr Cyril Bařinka), Jurkat, and RPMI 8226 (purchased from American Type Culture Collection-ATCC) were grown in full RPMI 1640 (Biowest). U251 and U2OS (ATCC) in full DMEM (Sigma). Capan-2 (ATCC) in McCoy's 5A (Gibco). HEK293 (ATCC) in EMEM (Sigma). CHO (ATCC) and PD-L1-transfected CHO cells (generated in our lab and briefly described below) were grown in full IMDM (supplemented by 4 mM L-glutamine and 25 mM HEPES).

Briefly, wild-type Chinese hamster ovary (WT CHO) cells were grown in a 24-well plate to reach approximately 50 to 60% confluency. A transfection solution of 0.5 μg pCMV3-full-length human PD-L1 plasmid (Sino Biological Inc cat# HG10084-CH) in 25 ml Opti-MEM (Gibco) and 1.5 ml lipofectamine (Invitrogen) was prepared and added to the well. The next day, cells were transferred to a 100 mm plate and grown in full IMDM medium containing 500 μg/ml hygromycin as a selection marker in the PD-L1 plasmid. The medium was changed every 3 days until the cell colonies were visible to the naked eye. At this point, a few colonies were picked, cultured separately, and tested for expression of membrane-bound PD-L1 with flow cytometry.

### Flow cytometry

To perform the experiment, the medium was removed, and cells were rinsed with 5 ml PBS (137 mM NaCl, 2.7 mM KCl, 13.5 mM K_2_HPO_4_, 5 mM NaH_2_PO_4_.2H_2_O, pH 7.2), then trypsinized with 1.5 ml trypsin/EDTA for 5 min at 37 °C, followed by addition of 6 ml medium to stop trypsinization. The cells were transferred into 15 ml tubes and centrifuged for 5 min at 300*g*. The medium was discarded, and the cell pellets were resuspended in 3 ml PBS. The cell number and viability (above 90%) were determined by an automated cell counter (Invitrogen). Then, 90 μl of cell suspension from each cell type (adjusted to 1.1 × 10^6^ cells/ml) was added into wells of a U-shaped polypropylene 96-well plate in duplicates and treated with 10 μl of 10 × dilution series of iBody 1 and iBody 3 for 30 min at 4 °C. Cells were washed twice with 200 μl PBS (5 min at 300*g*) and resuspended in 100 μl PBS. Finally, the cell suspension was measured with a BD LSRFortessa Flow Cytometer (Becton, Dickinson and Company) and analyzed with FlowJo 10.4.1. Staining of cells with ATTO488-WL12 was carried out using a similar procedure. The Gating strategy is explained in Fig. S7.

To stain the cell lines, cells were grown on 6-well plates in their corresponding full medium. Adherent cells were trypsinized for 5 min at 37 °C and washed twice with PBS along with suspension cells. The cell number and viability (above 85%) were measured using an automated cell counter (Invitrogen). From each cell line, 12 × 10^4^ cells per 100 μl PBS were transferred into wells of a U-shaped polypropylene 96-well plate. The plate was centrifuged (5 min at 250*g*) to discard PBS. Each well was stained with 100 μl of 100 nM iBody 1 dissolved in PBS, 100 μl of 1:100 FITC-α-PD-L1 antibody (clone 29E.2A3, Exbio cat# 1F-177-T100), 100 μl of 100 nM iBody 3, 100 μl of 100 nM control antibody (Ctrl Ab; mouse FITC-IgG2b, clone MPC-11, Exbio cat# 1F-692-C100), or 100 μl of PBS alone for 30 min at 4 °C. Next, cells were washed twice with 200 μl PBS (5 min at 300*g*) and resuspended in 100 μl PBS. Finally, the cell suspension was measured with a BD LSRFortessa Flow Cytometer (Becton, Dickinson and Company) and analyzed with FlowJo 10.4.1. Each cell population was gated based on their forward and side scatter properties, followed by gating for single cells. The Gating strategy is explained in Fig. S8.

### Confocal microscopy

PD-L1-transfected and wild-type CHO cells in 200 μl full IMDM medium (Biosera, 10% FBS, L-Glutamine, 25 mM HEPES) were seeded in a 96-well glass bottom microscopy plate (Cellvis; approximately 1000 cells per well) for 48 h at 37 °C. The medium was discarded, cells were washed twice with PBS and fixed with 75 μl 2% formaldehyde for 30 min at RT. Next, wells were blocked with 100 μl of 1% BSA (2 h, RT, shaker), followed by washing twice with 100 μl of 0.1% PBST (PBS with 0.1% Tween-20). Afterward, 10 nM of iBody 1, iBody 3, Alexa Fluor 488-conjugated-anti-hPD-L1 (Antibody; clone: MIH1, eBioscience cat# 53-5983-42) or control antibody (Ctrl Ab; Alexa Fluor 488-conjugated-mouse IgG1 kappa, clone: P3.6.2.8.1, eBioscience cat# 53-4714-80) in 50 μl/sample 0.1% PBST were added to corresponding wells (1 h, RT, shaker). Next, cells were washed four times, nuclei were stained with 75 μl (2 μg/ml) Hoechst 33342. Finally, imaging was carried out at RT using a Carl Zeiss microscope (LSM 780). For ATTO488 and Alexa Fluor 488, a 488 nm laser (max 25 mW) was set to 2% (detector gain 700 V). Emission was collected from 499 to 552 nm for pinhole 1 AU. For Hoechst 33342, a 405 nm laser (max 30 mW) was set to 2% (detector gain 700 V) and emission was collected from 412 to 490 nm for pinhole 1 AU. After taking all images with the same setup, further analysis was performed with ZEN 2.3 software.

### IL-2 and CD69 measurements in a cellular assay

Stable transfection of the Jurkat human leukemic T cell line with the human PD-1 gene and the Raji lymphoblastoid B cell line with the human PD-L1 gene have been described (30). Both cell lines were grown in full RPMI 1640 medium (Biosera, supplemented with 10% FBS, 1 mM sodium pyruvate, 2 mM glutamine, and 1x non-essential amino acids solution). On the day of the experiment, 50 × 10^3^ PD-L1-overexpressing Raji B cells in 50 μl full medium was added to wells of a U-shaped 96-well tissue culture plate for 1 h at 37 °C. Afterward, the cells were pre-incubated for 30 min at 37 °C with a dilution series of therapeutic antibodies (Selleckchem, atezolizumab cat# A2004, avelumab cat# A2015, durvalumab cat# A2013) as well as iBodies and WL12 were prepared in 100 μl/well full medium containing 50 pg/ml superantigen SEE (Toxin Technology, cat# ET404). Afterward, 50 × 10^3^ PD-1-overexpressing Jurkat T cells in 50 μl were added to all wells to co-culture with PD-L1-overexpressing Raji B cells for 24 h at 37 °C. The next day, supernatants were collected, and IL-2 levels were measured by a quantitative ELISA kit (R&D systems, cat# DY202-05) according to the manufacturer’s protocol. The IL-2 EC_50_ was calculated using a 4-parameter logistic function in GraphPad Prism 9.0.0.

Co-culture for analysis of CD69 expression was set up similarly as above, except that cells were incubated for 18 h in the presence of 2 nM of all compounds. Next, cells were collected by centrifugation (600*g*, 6 min) and washed once with 200 μl PBS. Corresponding wells were stained with 100 μl of 1:100 BV421-anti-human CD69 antibody (BD Biosciences, cat# 562884) or 100 ng/well isotype control antibody (BD Biosciences cat# 562438) in PBS for 30 min at 4 °C. Afterwards, cells were washed three times with 200 μl PBS followed by viability staining using 100 μl of 1:1000 Zombie NIR fixable viability kit (Biolegend, cat# 423106) in PBS. Finally, the measurement was carried out on an LSRFortessa Flow Cytometer (Becton, Dickinson and Company) and analyzed with FlowJo 10.4.1. The cell population was gated based on their forward and side scatter properties, followed by gating for single and viable cells. The Gating strategy is explained in the Fig. S9.

## Data availability

Further information and requests for resources and reagents generated in this study are available from the Lead Contact with a completed Materials Transfer Agreement.

## Supporting information

This article contains Supporting information.

## Conflict of interest

iBody technology is protected by patents US10114014 (B2) and US10302632 (B2). The authors declare that they have no conflicts of interest with the contents of this article.

## Supporting information

Supplementary methods, Supplemental Figures S1−S10 Legends and Table S1 Caption
