# Supplementary material for: Polymer-based antibody mimetics (iBodies) target human PD-L1 and function as a potent immune checkpoint blocker
Source: J Biol Chem. 2024 Apr 27;300(6):107325. doi: 10.1016/j.jbc.2024.107325 (PMC11154707; doi:10.1016/j.jbc.2024.107325)
Supplement: Supplementary methods, Supplemental Figures S1−S10 Legends and Table S1 Caption [file mmc1.docx]

**Supporting Information**Polymer-based antibody mimetics (iBodies) target human PD-L1 and function as a potent immune checkpoint blocker

Mohammad Reza Zamani^1,2^, Martin Hadzima^2,3^, Kristyna Blazkova^2^, Vladimír Šubr^5^, Tereza Ormsby^2^, Javier Celis-Gutierrez^4^, Bernard Malissen^4^, Libor Kostka^5^, Tomáš Etrych^5^, Pavel Šácha*^2^ and Jan Konvalinka*^2,6^

**Contents of Supporting Information:**

Supplemental figure S1: Structure of WL12.

Supplemental figure S2: Structure of ATTO488-WL12.

Supplemental figure S3: Structure of biotin-PEG-12-WL12.

Supplemental figure S4: HPLC chromatogram of WL12.

Supplemental figure S5: HPLC chromatogram of ATTO488-WL12.

Supplemental figure S6: HPLC chromatogram of biotin-PEG-12-WL12.

Supplemental figure S7: Gating used for CHO cell flow cytometry experiment.

Supplemental figure S8: Gating used for cell line flow cytometry experiment.

Supplemental figure S9: Gating used for CD69 detection with flow cytometry.

Supplemental figure S10: Reaction scheme of the iBody.

Supplemental Table 1. Characterization of copolymer precursors.

Supplementary methods:

Synthesis of linear WL12 precursor.

Cyclization of linear WL12 precursor.

Preparation of ATTO488-conjugated WL12.

Preparation of biotinylated WL12

Synthesis of polymer precursors and iBodies.

Characterization of polymer precursors and iBodies.

Supplement references.

**Supplementary methods**

**Sequence of WL12**

*cyclo*(AcTyr-MeAla-Asn-Pro-His-Leu-Hyp-Trp-Ser-Trp(Me)-MeNle-MeNle-Orn-Cys)-Gly-NH_2_

(Thioether bridge: Cys14-Ac-Tyr)

**Synthesis of linear WL12 precursor:**

*(*ClAc-Tyr-MeAla-Asn-Pro-His-Leu-Hyp-Trp-Ser-Trp(Me)-MeNle-MeNle-Orn-Cys-Gly-NH_2_*)*

Rink Amide MBHA resin (100-200 mesh) 0.71 mmol/g (0.28 g, 0.20 mmol) was left to swell in anhydrous dichloromethane (5 mL) for 10 min. The Fmoc group was removed by treatment with 20% piperidine in *N*,*N*-dimethylformamide (1 x 5 min, 1 x 30 min, 2 x 5 mL). Resin was washed with *N*,*N*-dimethylformamide (3 x 5 mL), 2-propanol (2 x 5 mL) and dichloromethane (3 x 5 mL). A solution of Fmoc-Gly-OH (0.30 g, 1.00 mmol, 5 eq.), HATU (0.38 g, 1.00 mmol, 5 eq.) and *N*,*N*-diisopropylethylamine (0.55 mL, 3.00 mmol, 15 eq.) in anhydrous *N*,*N*-dimethylformamide (5 mL) was added to the resin and the mixture was shaken for 16 h. Then, the resin was washed with *N*,*N*-dimethylformamide (2 x 5 mL), dichloromethane (2 x 5 mL), and *N*,*N*-dimethylformamide (3 x 5 mL). The Fmoc group was removed by treatment with 20% piperidine in *N*,*N*-dimethylformamide (1 x 5 min, 1 x 30 min, 2 x 5 mL). The resin was washed with *N*,*N*-dimethylformamide (3 x 5 mL), 2-propanol (2 x 5 mL) and dichloromethane (3 x 5 mL).

The peptide chain was extended following a general procedure for coupling of Fmoc-AA-OH using amino acids in the following order: Fmoc-Cys(Trt)-OH, Fmoc-Orn(Boc)-OH, Fmoc-MeNle-OH, Fmoc-MeNle-OH, Fmoc-Trp(Me)-OH, Fmoc-Ser(*t*Bu)-OH, Fmoc-Trp(Boc)-OH, Fmoc-Hyp(*t*Bu)-OH, Fmoc-Leu-OH, Fmoc-His(Trt)-OH, Fmoc-Pro-OH, Fmoc-Asn(Trt)-OH, Fmoc-MeAla-OH, Fmoc-Tyr(*t*Bu)-OH. The general procedure was as follows: a solution of Fmoc-AA-OH (1.00 mmol, 5 eq.), HATU (0.38 g, 1.00 mmol, 5 eq.) and *N*,*N*-diisopropylethylamine (0.55 mL, 3.00 mmol, 15 eq.) in anhydrous *N*,*N*-dimethylformamide (5 mL) was added to resin and the mixture was shaken for 3 h. Then, the resin was washed with *N*,*N*-dimethylformamide (2 x 5 mL), dichloromethane (2 x 5 mL) and *N*,*N*-dimethylformamide (3 x 5 mL). The Fmoc group was removed by treatment with 20% piperidine in *N*,*N*-dimethylformamide (1 x 5 min, 1 x 30 min, 2 x 5 mL). The resin was washed with *N*,*N*-dimethylformamide (3 x 5 mL), 2-propanol (2 x 5 mL), and dichloromethane (3 x 5 mL).

A few modifications were made in the case of Fmoc-Cys(Trt)-OH, for deprotection of secondary amines (MeNle, Hyp, Pro, MeAla) and for coupling to secondary amines. Coupling conditions for Fmoc-Cys(Trt)-OH (to limit racemization) were as follows: Fmoc-Cys(Trt)-OH (0.59 g, 1.00 mmol, 5 eq.), HATU (0.38 g, 1.00 mmol, 5 eq.), and 2,4,6-collidine (0.27 mL, 2.00 mmol, 10 eq.) in anhydrous *N*,*N*-dimethylformamide:dichloromethane / 1:1 (5 mL). Fmoc deprotection of secondary amines was conducted for 30 min + 40 min instead of 5 min + 30 min [2]. The coupling reaction to secondary amines was performed according to the general procedure for an extended amount of time, typically 16 h. Chloranil test was used to confirm completion of coupling reactions.

For final coupling of chloroacetic acid, a solution of chloroacetic acid (0.10 g, 1.00 mmol, 5 eq.), HATU (0.38 g, 1.00 mmol, 5 eq.), and 2,4,6-collidine (0.41 mL, 3.00 mmol, 15 eq.) in anhydrous *N*,*N*-dimethylformamide (5 mL) was added to resin and the mixture was shaken for 3 h. The resin was washed with *N*,*N*-dimethylformamide (2 x 5 mL) and dichloromethane (10 x 5 mL).

The product was cleaved from the resin by treatment with a 95:2.5:2.5 mixture of TFA:H_2_O:TIS (5 mL) for 1 h. The resin was filtered and washed with dichloromethane (4 x 5 mL). Volatiles were evaporated under reduced pressure, and the residue was triturated with Et_2_O (3 x 5 mL) to afford 300 mg of crude peptide material.

**Cyclization of linear WL12 precursor:**

The cyclization was performed according to a published procedure [3]. Briefly, the crude peptide was dissolved in 1:1 MeCN:0.1M NH_4_OAc (300 mL), and the pH was carefully adjusted to 8.5-9.0 using aq. 1 M NaOH. The solution was allowed to stand without stirring for 18 h. Solvents were subsequently removed by lyophilization. The product was purified by RP C18 HPLC (gradient 5-70% MeCN in H_2_O + 0.1% TFA) to obtain WL12 as a TFA salt (70 mg, 18%). MALDI MS: 1882 ([M + H]^+^). HR MALDI MS**:** Calculated. for C_91_H_129_O_20_N_22_S 1881.9474. Found 1881.9405.

**Preparation of ATTO488-conjugated WL12:**

WL12 (3.0 mg, 1.4 μmol) and *N*,*N*-diisopropylethylamine (20 μL, excess) were dissolved in *N*,*N*-dimethylformamide (1 mL). Then, ATTO488 NHS ester (1.0 mg, 1.4 μmol, 1.0 eq.) was added. The resulting mixture was stirred overnight at RT. Product was purified by RP C18 HPLC (gradient 5-70 % MeCN in H_2_O + 0.1% TFA) to obtain fluorescent WL-12 derivative modified with ATTO 488 as a TFA salt (2.0 mg, 53 %). Identity of the compound was verified by MALDI MS: 1226.0 ([M - H]^2-^). HR MALDI MS: Calculated . for C_116_H_150_O_29_N_25_S_3_ 2453.0194. Found 2453.0122.

**Preparation of biotinylated WL12 (Biotin-PEG-12-WL12)**

WL12 (4.0 mg, 1.9 μmol) and *N*,*N*-diisopropylethylamine (20 μL, excess) were dissolved in *N*,*N*-dimethylformamide (1 mL). Then, Biotin-PEG-12-NHS ester (2.1 mg, 2.3 μmol, 1.2 eq.) was added. The resulting mixture was stirred overnight at room temperature. The product was purified by RP C18 HPLC (gradient 15-50 % MeCN in H_2_O + 0.1% TFA) to obtain Biotin-PEG-12-WL12 derivative as a TFA salt (2.0 mg, 39 %). ESI MS: 1376.2 ([M + 2Na]^2+^). HR ESI MS: Calculated for C_128_H_195_O_35_N_25_Na_2_S_2_ 1376.17366. Found 1376.17310.

**Synthesis of polymer precursors and iBodies**

**Materials**

# 1-amino-propan-2-ol, methacryloyl chloride, β-alanin, 4,5 dihydrothiazole-2-thiol (TT), *N*-ethyl-*N*′-(3-dimethylaminopropyl)carbodiimide hydrochloride (EDC), carbon disulfide, ethanethiol, sodium hydride (60% dispersion in mineral oil), *N*,*N*-diisopropylethylamine (DIPEA), tert-butanol, *N*,*N*-dimethyl acetamide (DMAA) and dimethyl sulfoxide (DMSO) were purchased from Merck. HABA/Avidin Reagent kit was purchased from Sigma-Aldrich. NH_2_-(PEG)_11_-biotin was purchased from BroadPharm (Germany). Initiators 2,2′-azobis(2-methylpropionitrile) (AIBN) and 2,2′-azobis(4-methoxy-2,4-dimethylvaleronitrile) (V-70) were purchased from Wako Chemicals (Germany). ATTO488-amine was purchased from ATTO-TEC (Germany). Sephadex LH-20 and PD10 columns were purchased from APCzech (Czech Republic). All other chemicals and solvents were of analytical grade.

**Synthesis of polymer precursors**

Briefly, synthesis of precursor P1: 1.25 g (8.73 × 10^-3^ mmol) of HPMA (88 %mol), 0.308 g (1.19 × 10^-3^ mmol) of Ma-ß-Ala-TT (12 %mol) were dissolved in 2,0 ml *N*,*N*-dimethyl acetamide. 3.13 mg (1.53 × 10^-2^ mmol) of chain transfer agent S-2-cyano-2-propyl S′-ethyl trithiocarbonate, 2.35 mg (7.63 × 10^-3^ mmol) of initiator 2,2′-azobis(4-methoxy-2,4-dimethylvaleronitrile) (V-70) and 11.5 ml of tert-butanol were added. The solution of the polymerization mixture was introduced into a polymerization ampule. Before sealing the mixture was bubbled with argon for 10 min. Polymerization was carried out at 40 °C for 18 h. The polymer precursor was isolated by precipitation into a mixture of acetone:diethyl ether (3:1), filtered off, washed with acetone and diethyl ether and dried in vacuum. The terminating trithiocarbonate group was removed according to a procedure described by Perrier [4].

**Synthesis of iBody 1**

The copolymer precursor P1 (8 mg, 6.7 × 10^−3^ mmol TT groups), WL12 (2.2 mg, 1.04 × 10^−3^ mmol), NH_2_-PEG_11_-biotin (1.3 mg, 1.69 × 10^−3^ mmol) and ATTO488 (0.32 mg, 3.72 × 10^−4^ mmol) were dissolved in DMSO (300 μL), and then *N*,*N*-diisopropylethylamine (DIPEA) (2.7 μL, 3.1 ×10^−3^ mmol) was added. The reaction was carried out for 4 h at room temperature. Residual thiazolidine-2-thione reactive groups were removed by addition of 2 µl of 1-amino-propan-2-ol (2.59 × 10^−2^ mmol), and the reaction was stirred for next 10 min. The reaction mixture with iBody 1 was diluted with 1 mL of methanol and iBody 1 was separated on a 1.5×18 cm chromatography column with Sephadex LH-20 in methanol equipped with an UV-Vis detector Azura UVD 2.1S (Knauer). Methanol was evaporated, the iBody 1 was dissolved in Milli-Q water and purified on a PD10 column and lyophilized. The yield of the iBody 1 was 8 mg. The content of WL12 was 17.0 wt%; of ATTO488 was 2.3 wt%, of NH_2_-PEG_11_-biotin was 9.1 wt%; *M*_w_ = 122 000 g mol^-1^and *Ð* = 1.04.

**Synthesis of iBody 2**

The copolymer precursor P2 (7.1 mg, 5.98 × 10^−3^ mmol TT groups) and WL12, (2.84 mg, 1.35 × 10^−3^ mmol) was dissolved in DMSO (300 μL) and then *N*,*N*-diisopropylethylamine (DIPEA) (2.4 μL, 1.35 ×10^−3^ mmol) was added. The reaction conditions and purification are described above. The yield of the iBody 2 was 7 mg. The content of WL12 was 19.2 wt% and *M*_w_ = 85 000 g mol^-1^ and *Ð* = 1.13.

**Synthesis of iBody 3**

The copolymer precursor P1 (35 mg, 2.94 × 10^−2^ mmol TT groups), NH_2_-PEG_11_-biotin (6.3 mg, 1.72 × 10^−2^ mmol) and ATTO488 (1.60 mg, 1.86 × 10^−3^ mmol) were dissolved in DMSO (400 μL), and then *N*,*N*-diisopropylethylamine (DIPEA) (16.6 μL, 1.9 ×10^−2^ mmol) was added. The reaction conditions and purification are described above. The yield of the iBody 3 was 30 mg. The content of ATTO488 was 3.1 wt%, of NH_2_-PEG_11_-biotin was 12.5 wt%; *M*_w_ = 74 000 g mol^-1^and *Ð* = 1.06.

**Characterization of polymer precursors and iBodies.**

The weight-average molecular weights (*M*_w_), number-average molecular weights (*M*_n_), and dispersities (*Đ*) of the polymer precursors P1 and P2 and iBodies 1 – 3 were determined using a Shimadzu HPLC system equipped with a UV detector, an Optilab rEX differential refractometer, a DAWN 8 multiangle light scattering detector (Wyatt Technology, USA), and a TSKgel G4000SWXL size-exclusion chromatography column. The *M*_w_, *M*_n_ and *Đ* were calculated using Astra V software. The refractive index increment dn/dc = 0.167 ml/g was used for calculation. Mobile phase containing 300 mM sodium acetate buffer, pH 6.5, and methanol (20%/80% v/v). The flow rate was 0.5 ml/min. The content of TT reactive groups in the polymer precursors was determined spectrophotometrically (ε_305nm_ = 10,600 L mol^−1^ cm^−1^, methanol). The content of the fluorophore ATTO488 in the iBodies 1 and 2 (ε_502nm_ = 90,000 L mol^−1^ cm^−1^, water) was determined using spectrophotometr Specord 205 (Analytik Jena, Germany).

**Supplement references**

1. Zhou, X., et al., *First-in-Humans Evaluation of a PD-L1-Binding Peptide PET Radiotracer in Non-Small Cell Lung Cancer Patients.* J Nucl Med, 2022. **63**(4): p. 536-542.

2. Teixidó, M., F. Albericio, and E. Giralt, *Solid-phase synthesis and characterization of N-methyl-rich peptides.* J Pept Res, 2005. **65**(2): p. 153-66.

3. Miller, M.M., et al., *Macrocyclic inhibitors of the PD-1/PD-L1 AND CD80 (B7-1)/PD-L1 protein/protein interactions.WO2016039749A1, 2016.* .

4. Perrier, S., P. Takolpuckdee, and C.A. Mars, *Reversible addition-fragmentation chain transfer polymerization: End group modification for functionalized polymers and chain transfer agent recovery.* Macromolecules, 2005. **38**(6): p. 2033-2036.

**Supplemental figure and table legends**

Supplemental figure S1. Structure of WL12 [1]

Supplemental figure S2. Structure of ATTO488-WL12.

Supplemental figure S3. Structure of biotin-PEG-12-WL12.

Supplemental figure S4. HPLC chromatogram of WL12.

Supplemental figure S5. HPLC chromatogram of ATTO488-WL12.

Supplemental figure S6. HPLC chromatogram of biotinylated WL12.

Supplemental figure S7. Gating used for flow cytometry experiments in Figure 3 A and B. The cell population was gated based on their forward and side scatter properties, followed by gating for single cells.

Supplemental figure S8. Gating used for flow cytometry experiments in Figure 3 C. Each cell population was gated based on their forward and side scatter properties, followed by gating for single cells.

Supplemental figure S9. Gating used for flow cytometry experiments in Figure 4 C and D. The cell population was gated based on their forward and side scatter properties, followed by gating for single and viable cells.

Supplemental figure S10. Reaction scheme of the iBody. Polymerization reaction scheme followed by the polymer analogous conjugations of the desired moieties.

Table 1. Characterization of copolymer precursors.
