# Supplementary figures and images for: Polymer-based antibody mimetics (iBodies) target human PD-L1 and function as a potent immune checkpoint blocker
Source: J Biol Chem. 2024 Apr 27;300(6):107325. doi: 10.1016/j.jbc.2024.107325 (PMC11154707; doi:10.1016/j.jbc.2024.107325)

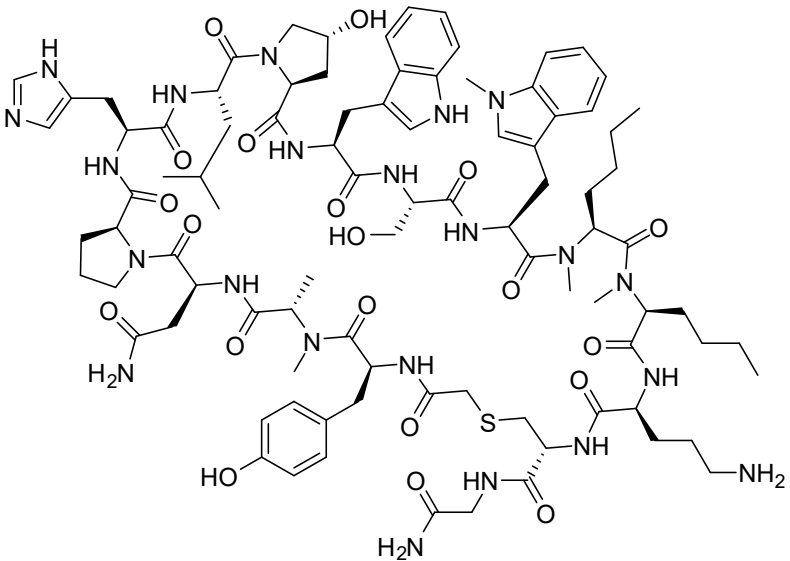

Supplement: Supplemental Figure S1 [file mmc2.pdf]

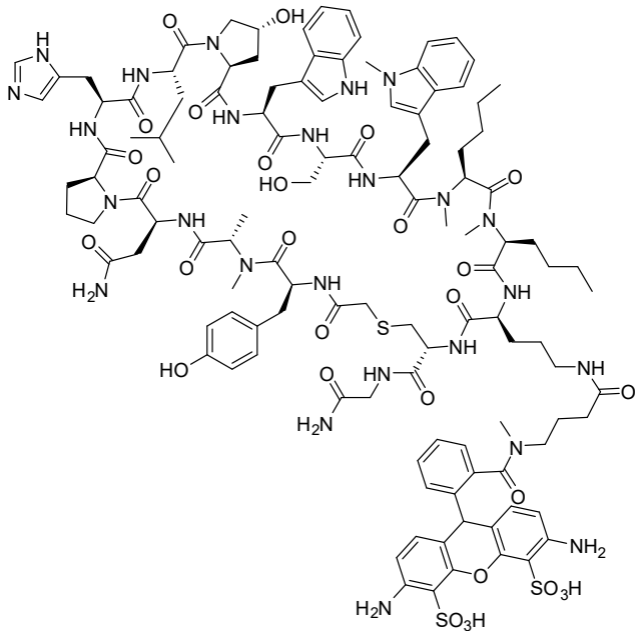

Supplement: Supplemental Figure S2 [file mmc3.pdf]

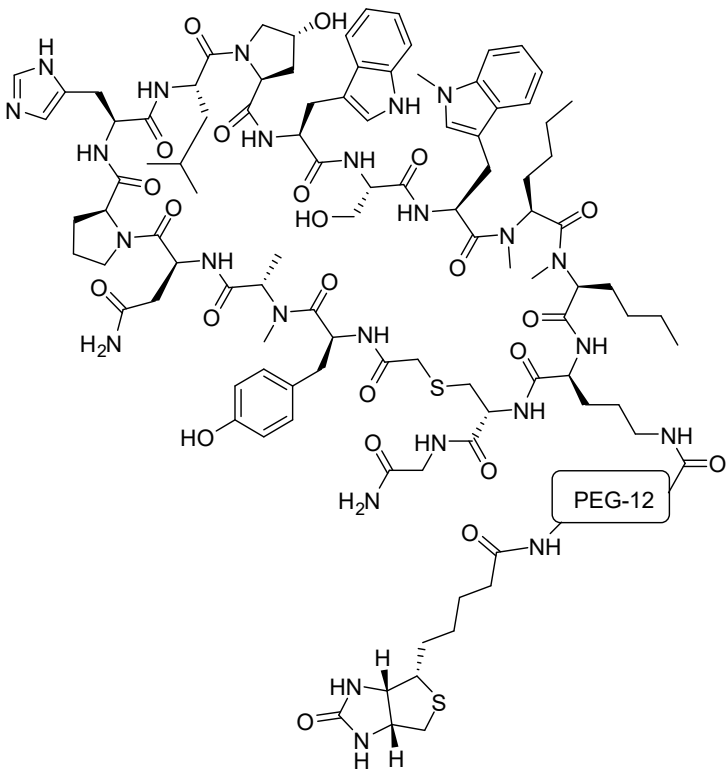

Supplement: Supplemental Figure S3 [file mmc4.pdf]

SSC-A

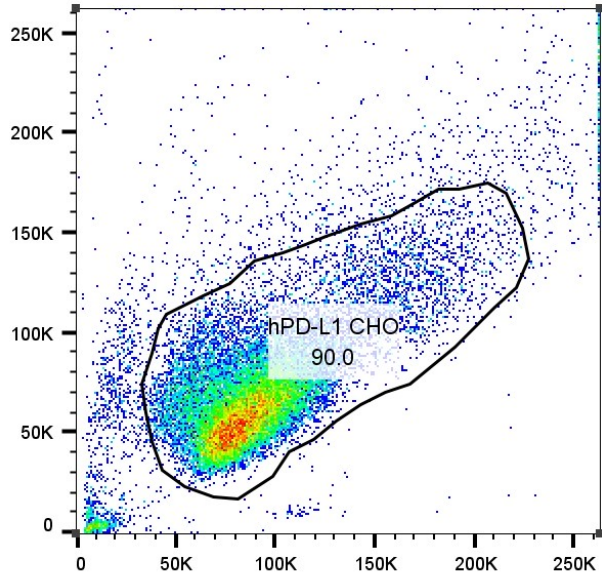

FSC-A

FSC-H

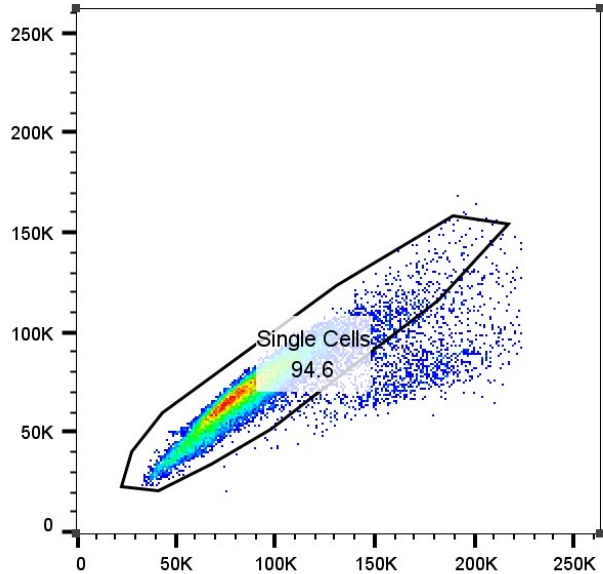

FSC-A

Supplement: Supplemental Figure S7 [file mmc8.pdf]

**Capan-2**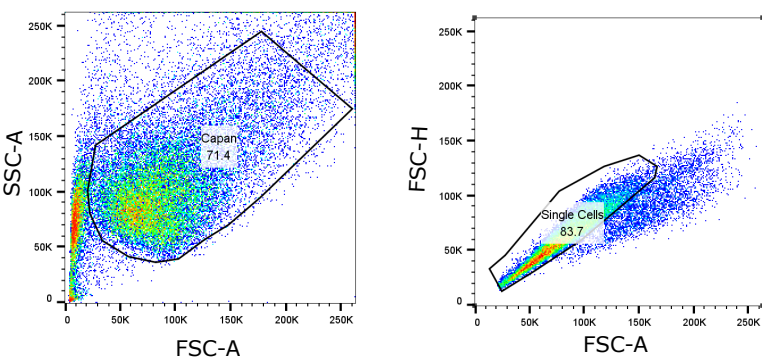**hPD-L1 CHO**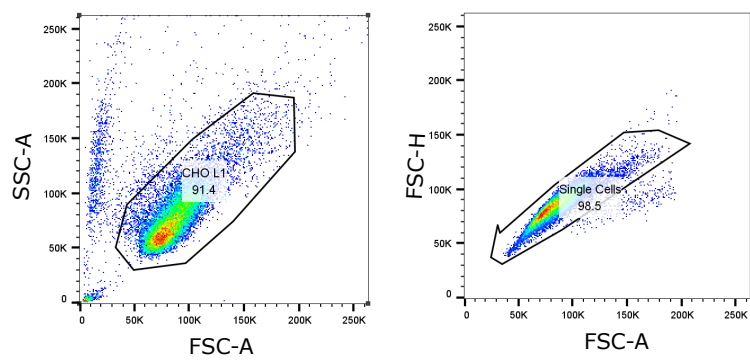**CHO**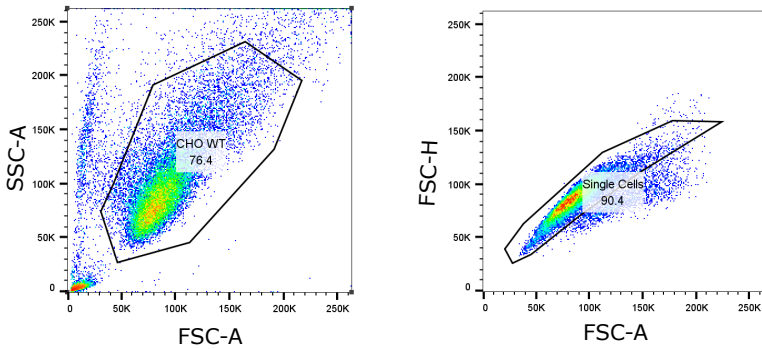**HEK293**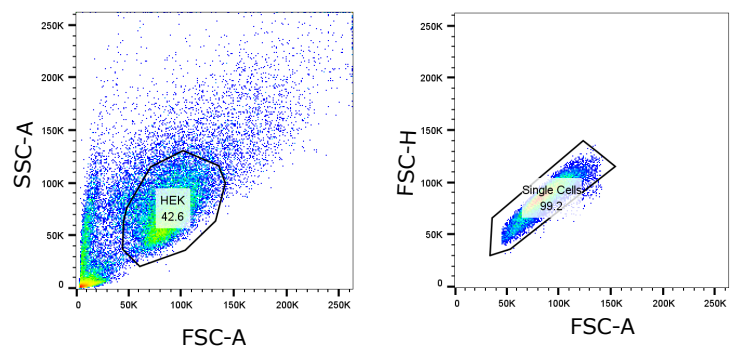**Jurkat**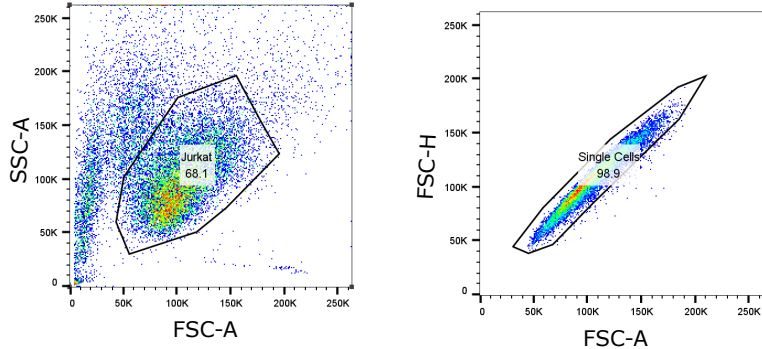**MDA-MB-231**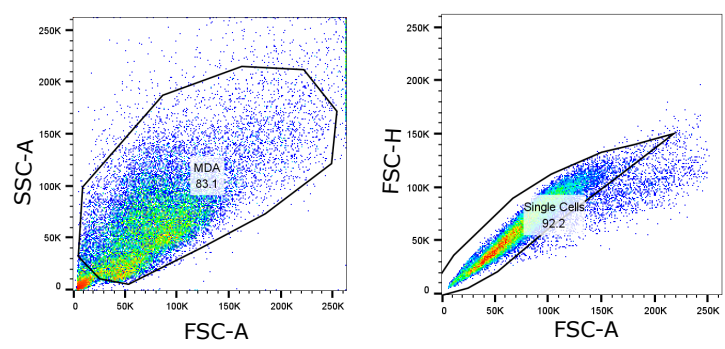**RPMI 8226**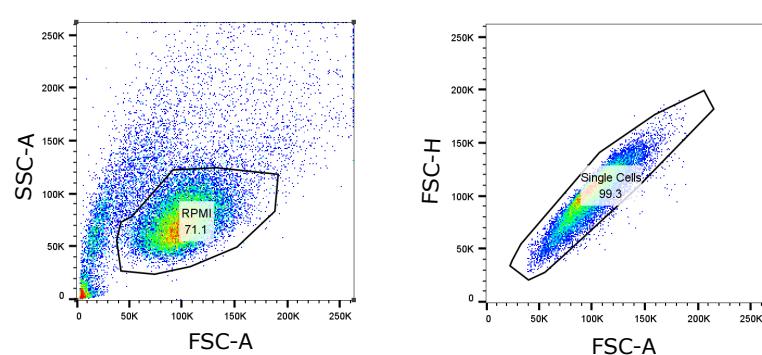**U2OS**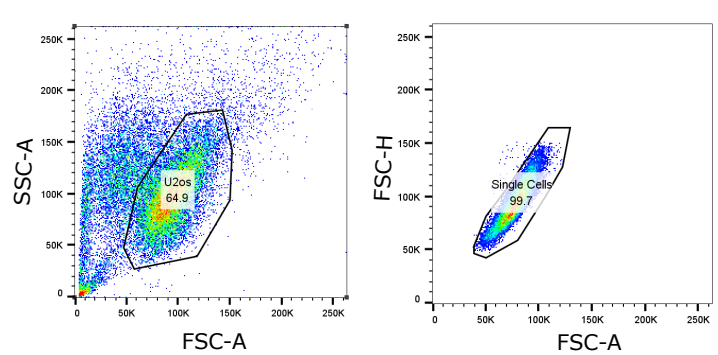**U251**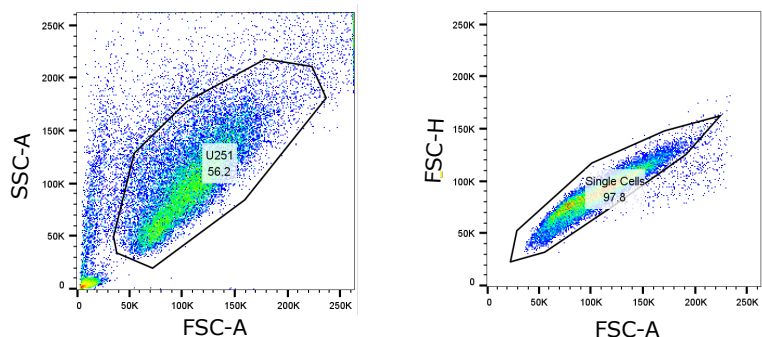

Supplement: Supplemental Figure S8 [file mmc9.pdf]

SSC-A

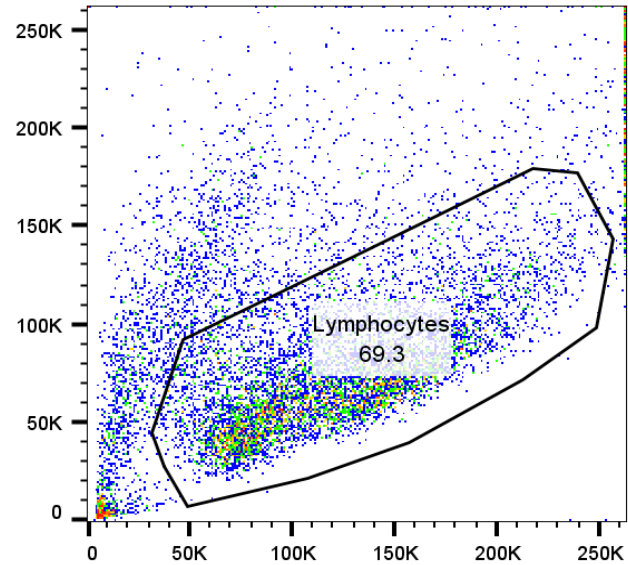

FSC-A

FSC-H

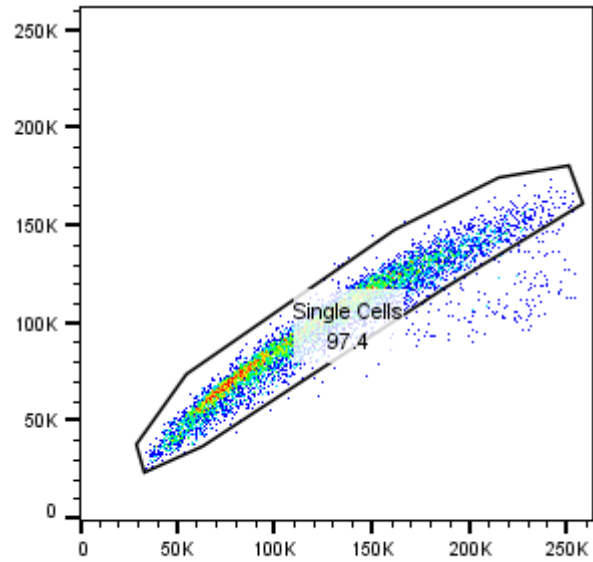

FSC-A

FSC-H

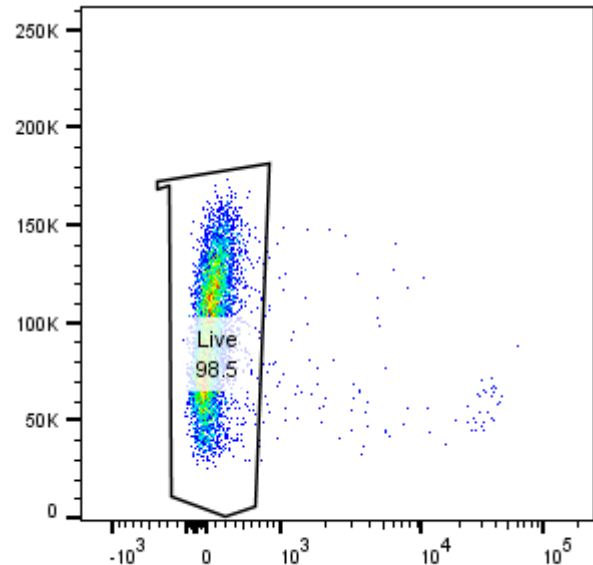

640-780\_60-A

Supplement: Supplemental Figure S9 [file mmc10.pdf]
