# Supplementary material for: Polymer-based antibody mimetics (iBodies) target human PD-L1 and function as a potent immune checkpoint blocker
Source: J Biol Chem. 2024 Apr 27;300(6):107325. doi: 10.1016/j.jbc.2024.107325 (PMC11154707; doi:10.1016/j.jbc.2024.107325)
Supplement: Supplemental Figure S5 [file mmc6.pdf]

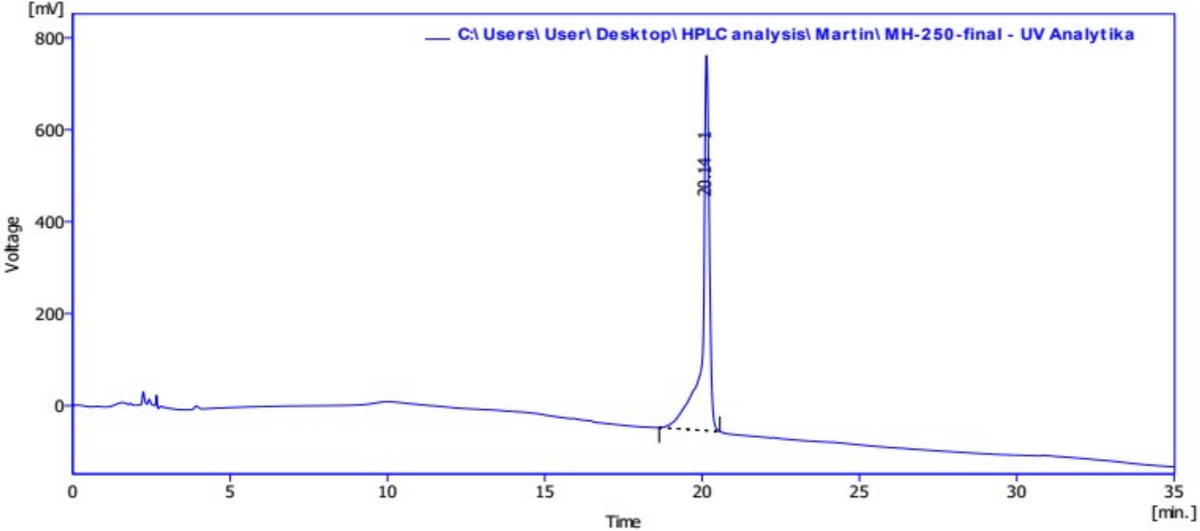

Result Table (Uncal - C:\Users\User\Desktop\HPLC analysis\Martin\MH-250-final - UV Analytika)

	Reten. Time [min]	Area [%]
1	20.140	100.0
	Total	100.0
