# Supplementary material for: Polymer-based antibody mimetics (iBodies) target human PD-L1 and function as a potent immune checkpoint blocker
Source: J Biol Chem. 2024 Apr 27;300(6):107325. doi: 10.1016/j.jbc.2024.107325 (PMC11154707; doi:10.1016/j.jbc.2024.107325)
Supplement: Supplemental Figure S6 [file mmc7.pdf]

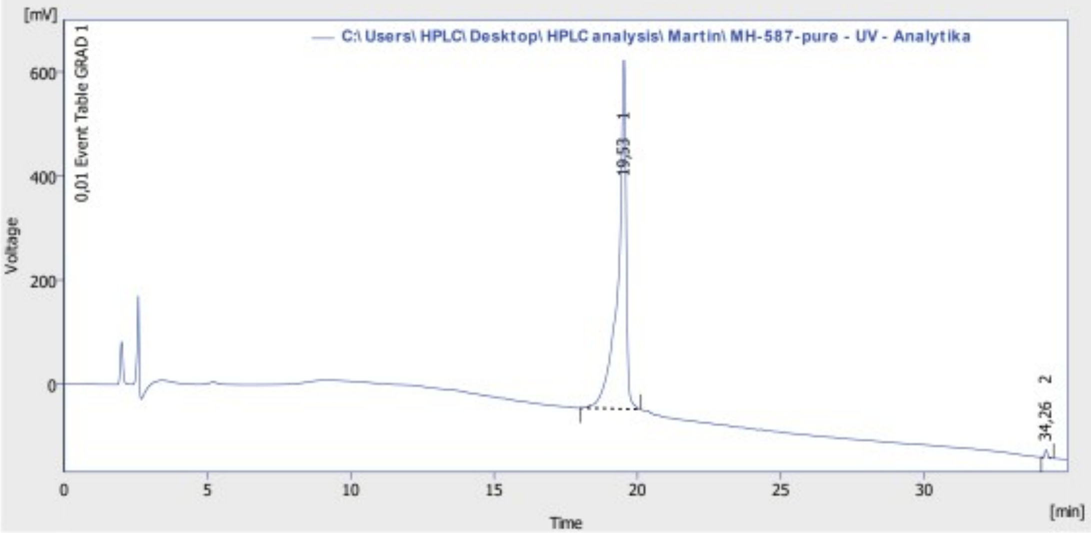

Result Table (Uncal - C:|Users|HPLC|Desktop|HPLC analysis|Martin|MH-587-pure - UV - Analytika)

	Reten. Time [min]	Area [mV.s]	Height [mV]	Area [%]	Height [%]	W05 [min]	Compound Name
1	19,529	13044,694	669,696	99,3	97,8	0,21	
2	34,256	95,338	15,241	0,7	2,2	0,10	
	Total	13140,032	684,937	100,0	100,0		
