# Supplementary material for: Polymer-based antibody mimetics (iBodies) target human PD-L1 and function as a potent immune checkpoint blocker
Source: J Biol Chem. 2024 Apr 27;300(6):107325. doi: 10.1016/j.jbc.2024.107325 (PMC11154707; doi:10.1016/j.jbc.2024.107325)
Supplement: Supplemental Figure S10 [file mmc11.pdf]

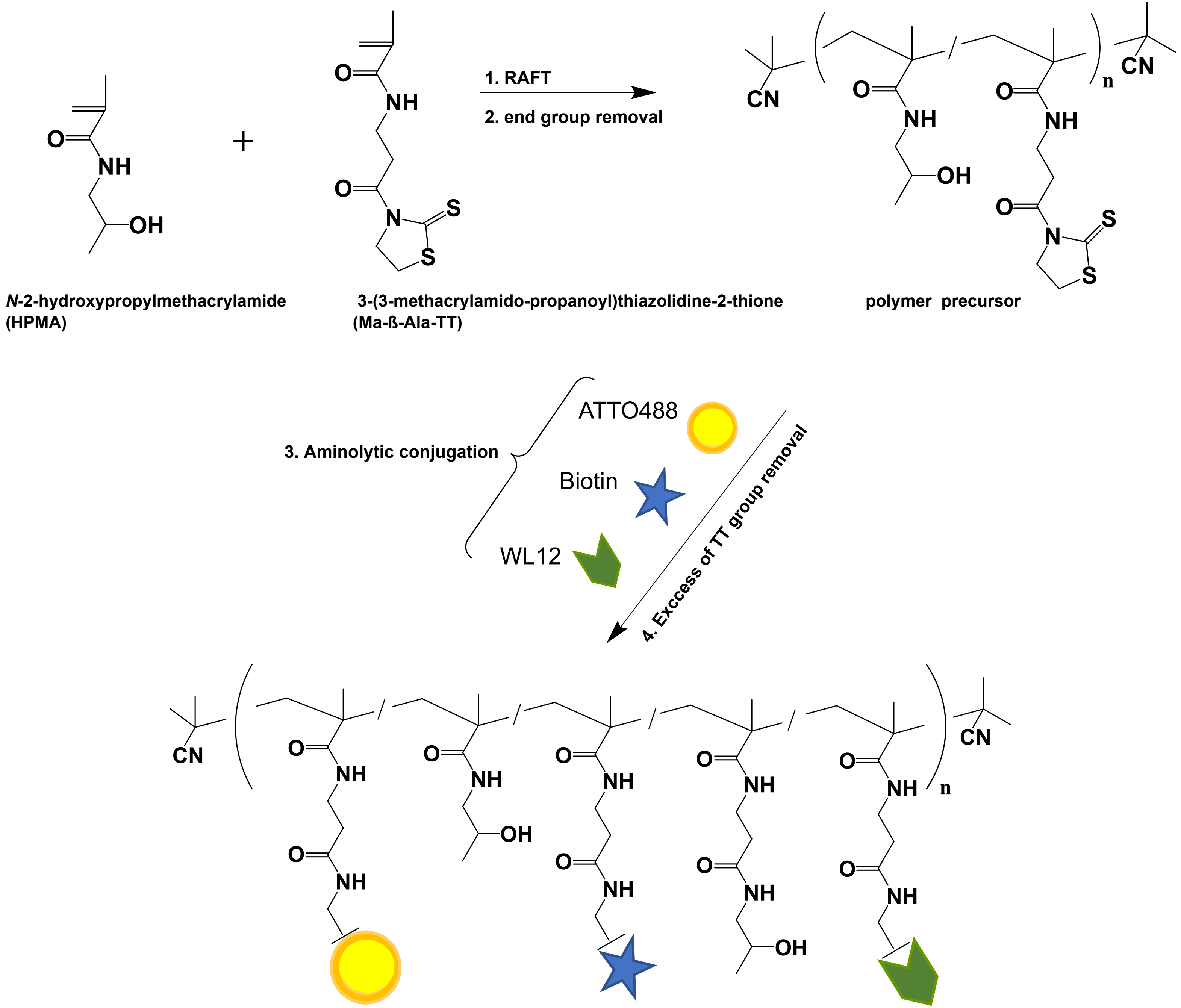
1. RAFT
2. end group removal
N-2-hydroxypropylmethacrylamide (HPMA)
3-(3-methacrylamido-propanoyl)thiazolidine-2-thione (Ma-ß-Ala-TT)
polymer precursor
3. Aminolytic conjugation
ATTO488
Biotin
WL12
4. Exccess of TT group removal
