# Supplementary material for: Polymer-based antibody mimetics (iBodies) target human PD-L1 and function as a potent immune checkpoint blocker
Source: J Biol Chem. 2024 Apr 27;300(6):107325. doi: 10.1016/j.jbc.2024.107325 (PMC11154707; doi:10.1016/j.jbc.2024.107325)
Supplement: Supplemental Table S1 [file mmc12.docx]

| Precursor | *M*_w_  g.mol^-1^ | *M*_n_  g.mol^-1^ | *Ð* | TT reactive groups  % mol |
| --- | --- | --- | --- | --- |
| P1 | 62 000 | 59 000 | 1.05 | 13.3 |
| P2 | 66 000 | 56 000 | 1.18 | 14.1 |
